# Supplementary material for: Notch signalling governs human enteric nervous system progenitor dynamics
Source: Development. 2026 Oct 23;153(20):dev205775. doi: 10.1242/dev.205775 (PMC13723397; doi:10.1242/dev.205775)
Supplement: Supplementary file 1 [file develop-153-205775-s1.pdf]

Figure S1

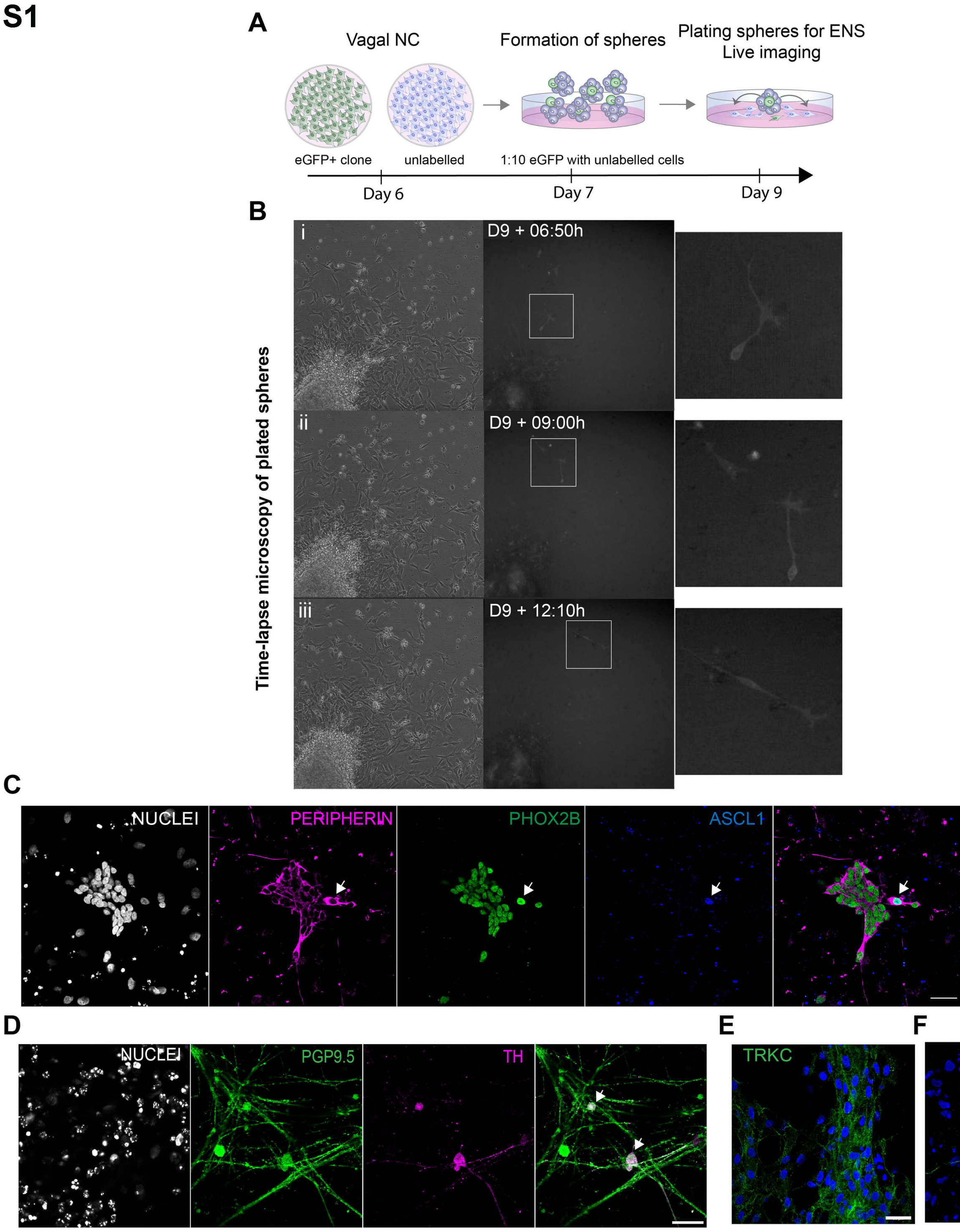

Figure S1. Emergence of human enteric neurons in vitro. (A) Schematic of the experimental strategy used to monitor ENS differentiation following sphere plating. GFP-expressing (Rainbow WTC-11) and unlabelled (WTC11) cells were differentiated to vagal NC and mixed at a 1:10 ratio prior to sphere formation. On day 9, spheres were plated under ENS-inducing conditions and live-imaged to track migration of GFP+ progenitor cells.
(B) Representative snapshots from time-lapse microscopy (brightfield and fluorescence) showing neurons migrating out of spheres at the indicated hours (h) after plating on day 9 (D9). Insets show magnified areas. Images were obtained every 10 minutes for 75 hours. (C) Immunofluorescence analysis of ASCL1, PHOX2B and PERIPHERIN in day 50 ENS cultures. The arrow indicates a triple-positive cell. (D-F) Immunofluorescence analysis of the indicated enteric neuronal markers in day 50 ENS cultures. Arrows indicate double-positive cells. Scale bars: 30 μm.

Development • Supplementary information

Figure S2

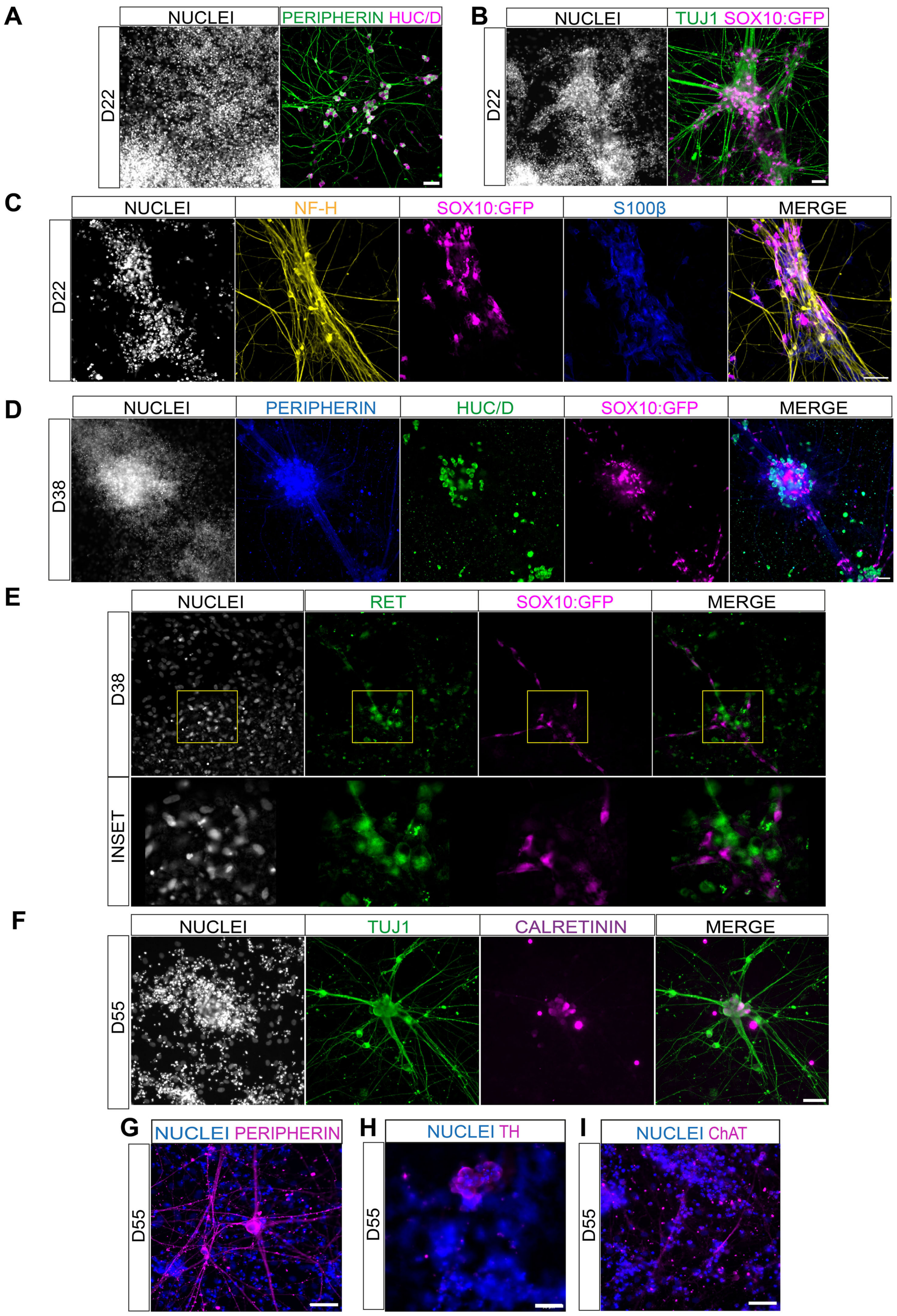

Figure S2. In vitro differentiation of SOX10:GFP hESCs toward ENS cell types. Immunofluorescence analysis of hESC-derived ENS cultures generated using the H9SOX10:GFP reporter cell line. (A-C) Expression of PERIPHERIN, HUC/D (A) TUJ1, SOX10:GFP (B) and NF-H, SOX10:GFP and S100β (C) in day 22 cultures. (D, E) Expression of PERIPHERIN, HUC/D, SOX10:GFP (D) and RET, SOX10:GFP (E) in day 38 cultures. Insets show magnified regions. (F-I) Expression of TUJ1 and the neural subtype marker CALRETININ (F), PERIPHERIN (G), TH (H) and ChAT (I) in day 55 ENS cultures. Scale bars=50 µm. D, days.

Development • Supplementary information

Figure S3

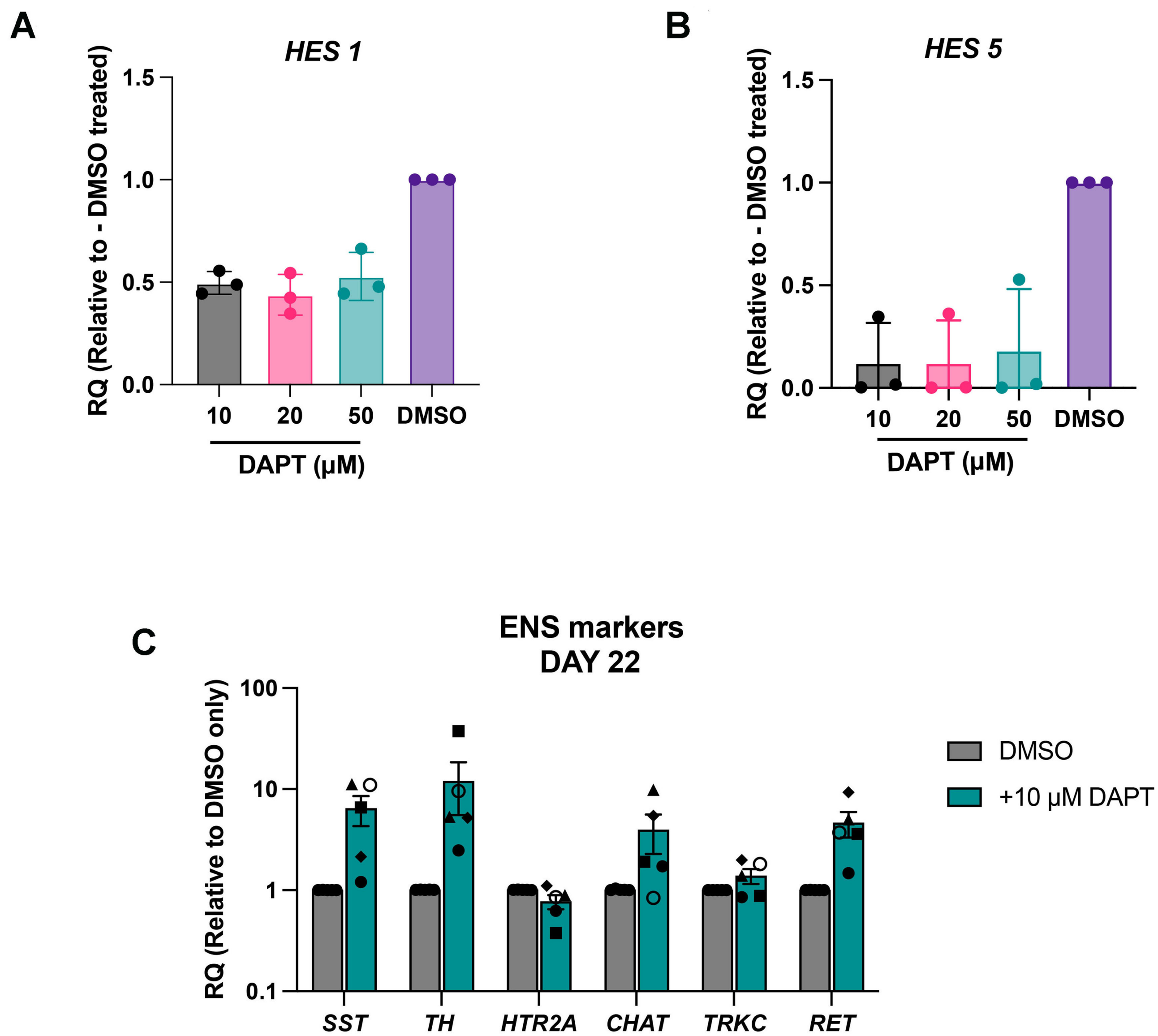

Figure S3. Impact of Notch inhibition on ENS progenitor differentiation. qPCR analysis of *HES1* (A) and *HES5* (B) expression in day 22 ENS cultures derived from H9SOX10:GFP hESCs and treated with increasing concentrations of DAPT (10, 20, and 50 μM) or a vehicle control (DMSO, matched to the 50 μM DAPT treatment). Relative gene expression was normalised to the vehicle control (set to 1). Data are presented as mean ± s.d. from three biological replicates (n = 3). (C) qPCR analysis of indicated ENS markers in day 22 cultures. Data are shown as mean values ± s.e.m (n=5). Different symbols indicate independent experiments. Statistical analysis: two-way ANOVA followed by Sidak's post-hoc multiple comparisons test; *P <0.05, **P <0.01, ***P<0.001. Only statistically significant changes are indicated.

Figure S4

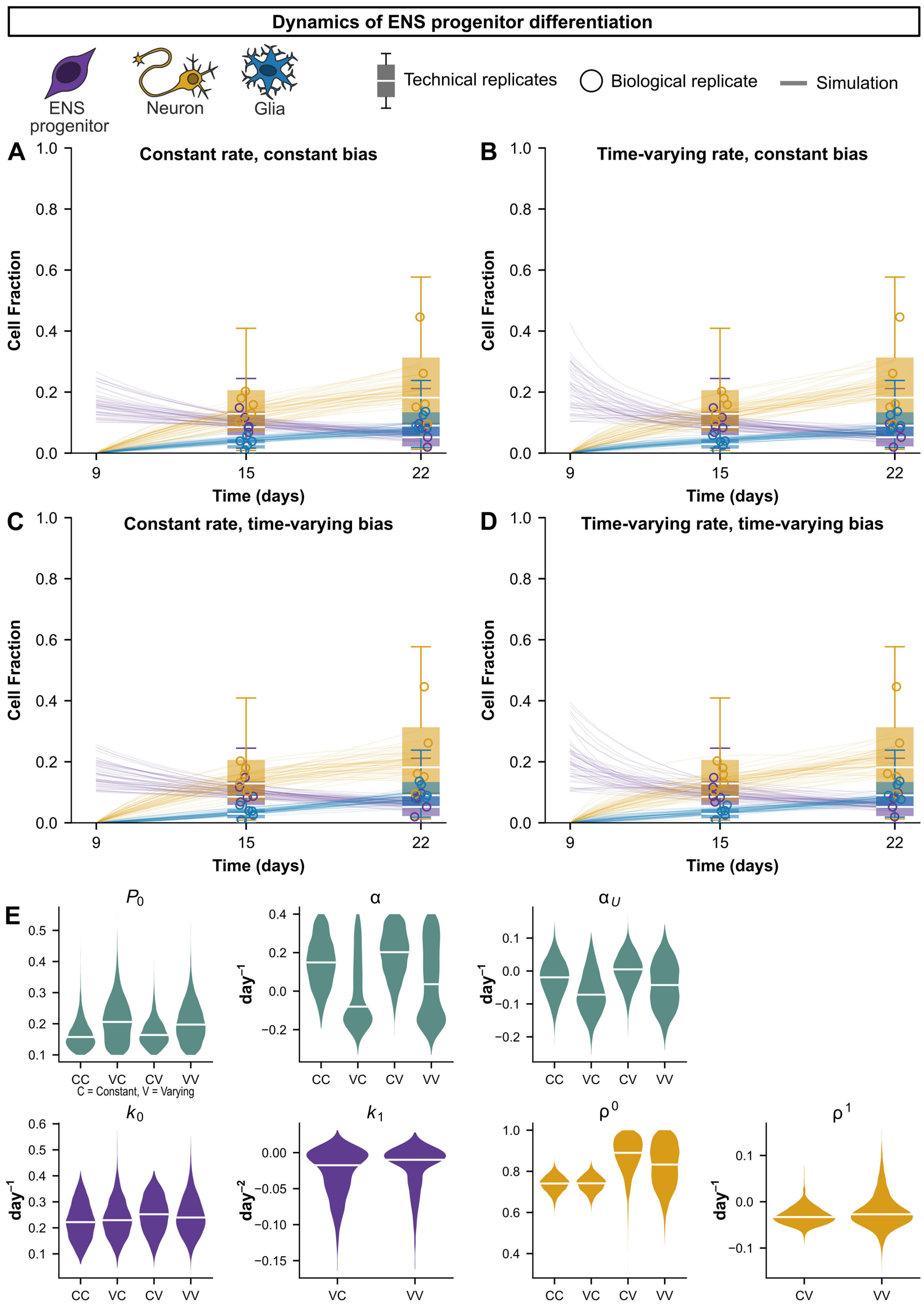

Figure S4. Posterior predictive checks and parameter distributions for ENS progenitor differentiation in DMSO control conditions.(A-D) Numerical simulations (lines) of progenitor (purple), neuronal (yellow), and glial (blue) fractions for models where differentiation rate is constant (A, C) or time-varying (B, D) and lineage bias is constant (A, B) or time-varying (C, D), respectively, using 50 samples drawn from the posterior distribution inferred from empirical data on ENS progenitor differentiation dynamics (markers and boxplots; see also Fig 4E, G , DMSO). Open circles indicate biological replicates, box plots show the median and interquartile range of the technical replicate data, and lines denote simulations. (E) Posterior marginal distributions of inferred parameters for the initial progenitor fraction P_0, the net per-capita growth rate of ENS progenitors α, the net per-capita rate of the unidentified population α_U, the initial differentiation rate k^0, the differentiation rate gradient k^1, the initial lineage bias ρ, and the neuronal lineage bias gradient, ρ^1.

Development • Supplementary information

Figure S5

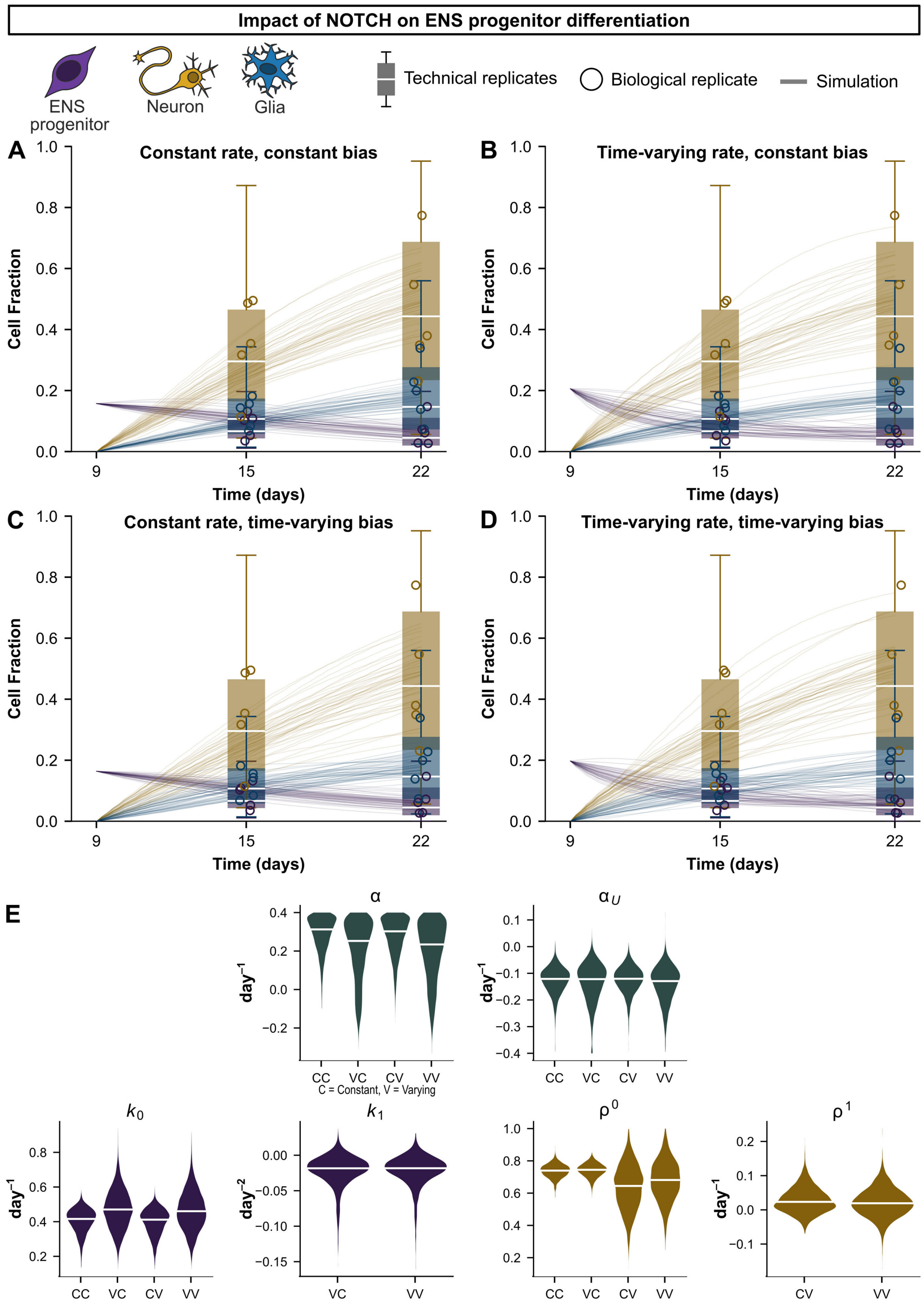

Figure S5. Posterior predictive checks and parameter distributions for ENS progenitor differentiation in DAPT treatment conditions. (A-D) Numerical simulations (lines) of progenitor (purple), neuronal(yellow), and glial (blue) fractions for models where differentiation rate is constant (A, C) or time-varying (B, D) and lineage bias is constant (A, B) or time-varying (C, D), respectively, using 50 samples drawn from the posterior distribution inferred from empirical data on ENS progenitor differentiation dynamics under NOTCH inhibition (markers and boxplots; (see also Fig 4E, G, DAPT). Open circles indicate biological replicates, box plots show the median and interquartile range of the technical replicate data, and lines denote simulations. (E) Posterior marginal distributions of inferred parameters: the net per-capita growth rate of ENS progenitors α, the net per-capita rate of the unidentified population α_U, the initial differentiation rate k^0, the differentiation rate gradient k^1, the initial lineage bias ρ, and the neuronal lineage bias gradient ρ^1.

Figure S6

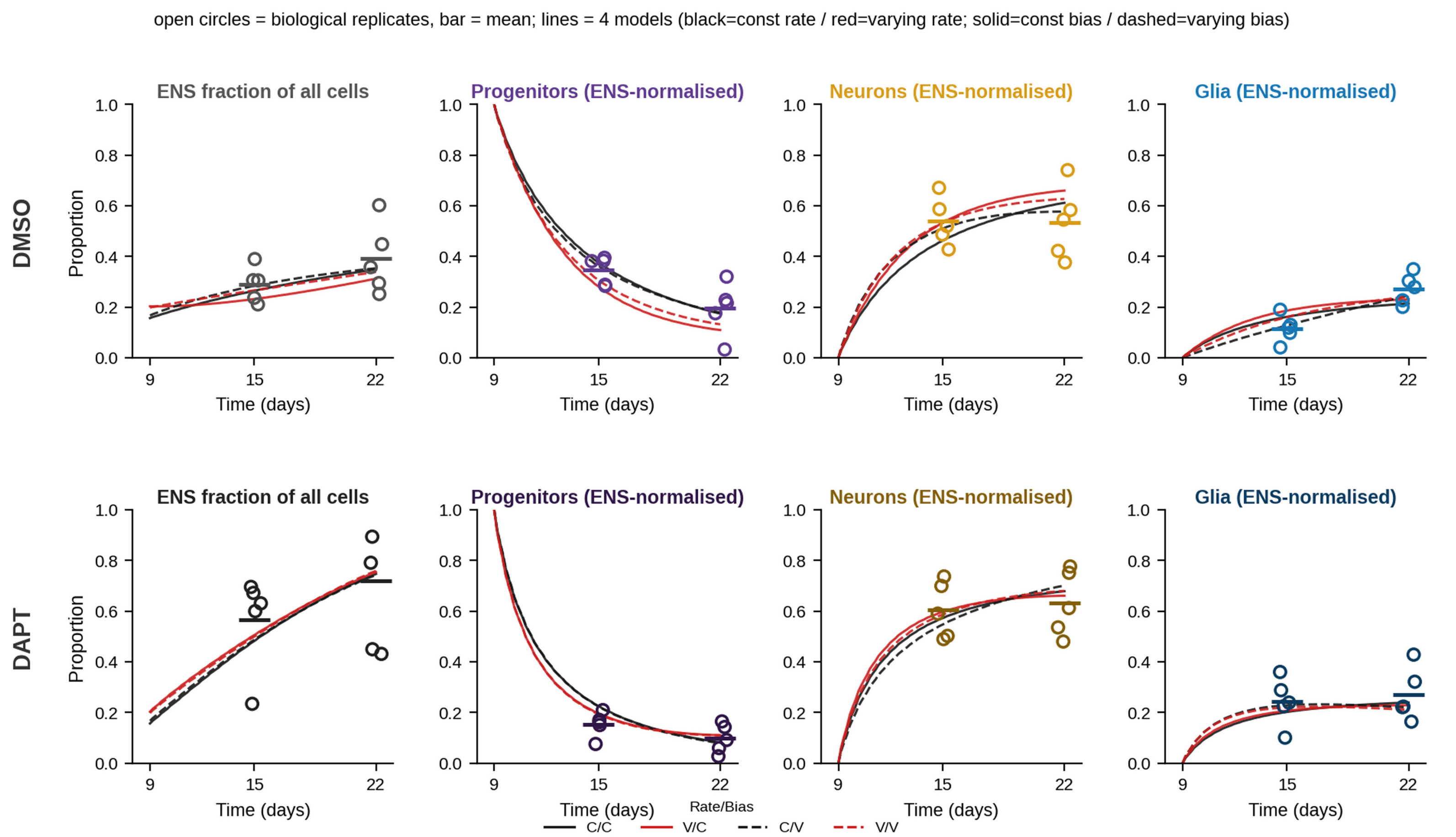

Figure S6. Cell type proportions normalised to the ENS compartment under control and Notch-inhibited conditions. Proportions of ENS cell types expressed relative to the ENS compartment alone (progenitors + neurons + glia), rather than to all cells scored, for DMSO control (top row) and DAPT treatment (bottom row). Leftmost panels show the ENS compartment as a fraction of all cells; remaining panels show progenitor (purple), neuronal (yellow) and glial (blue) proportions within that compartment. Open circles indicate biological replicates, obtained by combining the HuC/D and SOX10/S100β immunofluorescence fields for each replicate; horizontal bars denote their means. Lines denote simulations using median posterior parameter values for models where differentiation rate is constant (black) or time-varying (red) and lineage bias is constant (solid) or time-varying (dashed); see also Fig. 4E, G. Because the unidentified population is dynamically uncoupled from the ENS lineages, these normalised proportions are independent of P_0 and α_U, and are obtained from the same posterior distributions as Fig. 5 without refitting.

Figure S7

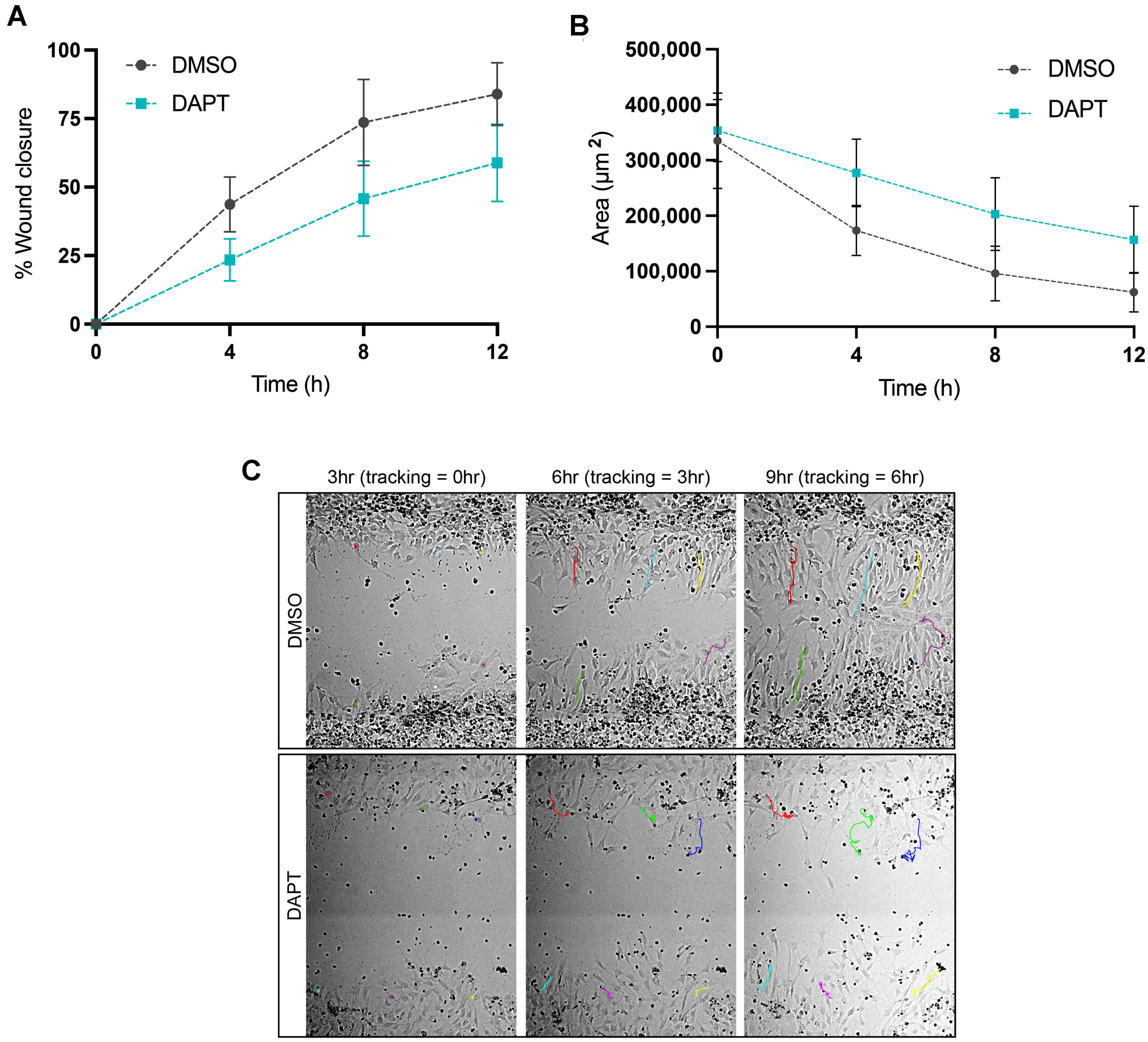

Figure S7. Effect of Notch inhibition on human ENS progenitor migration. (A) Percentage of closure area. Data are presented as mean ± s.e.m, n=3 independent experiments with 3-5 fields scored per condition. Fields were sampled from 1-3 wells per experiment. (B) Scratch area in μm2. Data are presented as mean ± s.e.m, n=3 independent experiments with 3-5 fields scored per condition. Fields were sampled from 1-3 wells per experiment . (C) Representative brightfield time-lapse images of DMSO and DAPT treated ENS cultures captured at 3, 6 and 9 hour hours post-wounding. Coloured lines/dot show individual tracked cell trajectories.

Development • Supplementary information

Table S1. Summary of inferred model parameters. Posterior median and 90% credible interval [5th–95th percentile] for each parameter under the four differentiation scenarios (CC, CV, VC, VV), fitted to control (DMSO) and Notch-inhibited (DAPT) data by the No-U-Turn sampler. Parameters: P_0, initial progenitor fraction at D9; α, net progenitor proliferation rate (day^{-1}); α_U, net rate of the unidentified population (day^{-1}); k_0, initial differentiation rate (day^{-1}); k_1, differentiation-rate gradient (day^{-2}; time-varying-rate models only); ρ^0, initial neuronal lineage bias; ρ^1, neuronal-bias gradient (day^{-1}; time-varying-bias models only); φ_N, φ_{PG}, field-level overdispersion (concentration) parameters for the neuron and SOX10/S100β fields. Dashes denote parameters not present in a given scenario. For DAPT, P_0 was fixed to the DMSO-inferred value, since the cultures are identical at D9 before treatment (see Methods).

DMSO (control)

Parameter	CC	CV	VC
P_0	0.157 [0.109, 0.250]	0.164 [0.113, 0.251]	0.206 [0.112, 0.335]
α	+0.149 [-0.063, +0.357]	+0.202 [-0.019, +0.375]	-0.081 [-0.214, +0.329]
α_U	-0.019 [-0.117, +0.065]	+0.005 [-0.086, +0.079]	-0.072 [-0.163, +0.048]
k_0	0.222 [0.112, 0.347]	0.252 [0.134, 0.363]	0.229 [0.124, 0.362]
k_1	—	—	-0.018 [-0.092, +0.011]
ρ^0	0.741 [0.672, 0.800]	0.890 [0.714, 0.985]	0.742 [0.673, 0.800]
ρ^1	—	-0.033 [-0.057, +0.002]	—
φ_N	27 [10, 56]	30 [12, 61]	27 [11, 58]
φ_{PG}	80 [41, 149]	93 [46, 167]	81 [42, 147]

DAPT (Notch inhibition)

Parameter	CC	CV	VC
P_0	0.157 (fixed)	0.164 (fixed)	0.206 (fixed)
α	+0.312 [+0.114, +0.393]	+0.302 [+0.083, +0.392]	+0.253 [-0.105, +0.388]
α_U	-0.121 [-0.215, -0.058]	-0.120 [-0.213, -0.053]	-0.122 [-0.264, -0.040]
k_0	0.416 [0.278, 0.498]	0.412 [0.261, 0.499]	0.470 [0.295, 0.652]
k_1	—	—	-0.018 [-0.065, +0.005]
ρ^0	0.740 [0.670, 0.800]	0.644 [0.406, 0.854]	0.745 [0.670, 0.804]
ρ^1	—	+0.023 [-0.026, +0.093]	—
φ_N	6 [3, 11]	6 [3, 11]	6 [3, 11]
φ_{PG}	31 [17, 53]	32 [17, 54]	32 [16, 54]

Development • Supplementary information
