# Supplementary material for: Notch signalling governs human enteric nervous system progenitor dynamics
Source: Development. 2026 Oct 23;153(20):dev205775. doi: 10.1242/dev.205775 (PMC13723397; doi:10.1242/dev.205775)
Supplement: Supplementary file 2 [file develop-153-205775_review_history.pdf]

Notch signalling governs human enteric nervous system progenitor dynamics

Antigoni Gogolou, Nikolas Stefanidis, Fay Cooper, Guillaume Blin, Stanley E. Strawbridge, Alexander G. Fletcher and Anestis Tsakiridis
DOI: 10.1242/dev.205775

Editor: James M Wells

Review timeline
Original submission: 30 April 2026
Editorial decision: 14 June 2026
First revision received: 24 August 2026
Accepted: 21 September 2026

Original submission

First decision letter

MS ID#: dev.205775

MS TITLE: Notch signalling governs human enteric nervous system progenitor dynamics

AUTHORS: Anestis Tsakiridis; Antigoni Gogolou; Nikolas Stefanidis; Fay Cooper; Guillaume Blin; Stanley E. Strawbridge; Alexander G. Fletcher

Dear Dr Tsakiridis,

I have now received all the referees' reports on the above manuscript, and have reached a decision. The referees' comments are appended below.

As you will see, the referees express considerable interest in your work, but have some significant criticisms and recommend a substantial revision of your manuscript before we can consider publication. If you are able to revise the manuscript along the lines suggested, which may involve further experiments, I will be happy receive a revised version of the manuscript. Your revised paper will be re-reviewed by one or more of the original referees, and acceptance of your manuscript will depend on your addressing satisfactorily the reviewers' major concerns. Please also note that Development will normally permit only one round of major revision. If it would be helpful, you are welcome to contact us to discuss your revision in greater detail. Please send us a point-by-point response indicating your plans for addressing the referees' comments, and we will look over this and provide further guidance.

Please attend to all of the reviewers' comments and ensure that you upload both a 'clean' version of your Word file, along with a highlighted version clearly showing where you have made changes in the revised manuscript. Please avoid using 'Tracked changes' in Word files as these are lost in PDF conversion. I should be grateful if you would also provide a point-by-point response detailing how you have dealt with the points raised by the reviewers in the 'Response to Reviewers' box. If you do not agree with any of their criticisms or suggestions please explain clearly why this is so.

Reviewer 1

© 2026. Published by The Company of Biologists under the terms of the Creative Commons Attribution License (https://creativecommons.org/licenses/by/4.0/).

This is an interesting study using human pluripotent stem cell (hPSC) models of the ENS to study the impact of Notch signalling on neurogenic and gliogenic cell fates. Here the authors combine gene expression analysis, immunohistochemistry and quantitative computational modelling to examine the emergence of neural and glial progenitors in the presence or absence of potent Notch signalling inhibitor DAPT. They find inhibition of Notch results in premature or accelerated differentiation, to both neuronal and glial cell lineages. Further, scratch assay suggests migratory capacity of ENS progenitors is also altered. Comments to authors below:

1. The authors themselves describe the culture conditions (that spheres are plating into) as "neurogenic conditions". How does this impact the conclusions drawn about both the differentiation trajectories in normal conditions, and the impact of Notch signalling? Can Notch inhibition drive neuronal and glial differentiation of vNCC/ENCC in the absence of neurogenic conditions (GDNF, AA)?
2. In Figure 3B, significant upregulation of DLL1/3/4 in the Notch inhibition conditions makes sense. But can the authors explain the significant upregulation of Notch 2/Jag 2 after DAPT treatment (at later timepoints)?
3. Line 235-237: I think "…throughout the course of differentiation" slightly misrepresents the data. It suggests all transcripts upregulated across all (or most) time points, however most genes only significantly upregulated at 1-2 time points. Maybe rephrase (eg: "….at different time points throughout the course of differentiation".
4. The scratch assay data is compelling. But I find it a little difficult to separate a direct effect on migration from the accelerated differentiation associated with DAPT treatment. Could this reduced wound closure be purely a symptom of less progenitors and more differentiation, rather than actual migration? Have you looked at expression of key migratory genes following DAPT treatment?
5. One of the main conclusions (in the abstract) is that inhibition of Notch "…accelerates differentiation coincident with a shift toward increased gliogenesis". I don't see strong evidence from the biology of a shift toward glial fate. The modelling may suggest it, but I think if that's the only evidence then the language needs to be softened or the sentence clarified.

Minor:

- Line 90: Already a number of references included, but would consider adding McCallum et al 2020 (PMID: 32851974) as this directly addresses the role of Notch signalling in enteic neuro-gliogenesis (in zebrafish).
- Figure 4: Please label the nearest neighbour quantification (bar graph) Figure 4J, for consistency.

<u>Reviewer 2</u>

This study explores a very timely application of in vitro models to the investigation of Notch signalling-based regulation of the ENS development. The authors utilise their previously published approach for stem cell-based derivation of ENS lineages and proceed to characterise this model as well as perturb it via DAPT-mediated Notch inhibition. Findings from this work are evaluated through quantitative modelling and scratch assay.

Overall, the work presented is timely and paves the way for the use of this type of in vitro systems in ENS research. The characterisation of the iPSC-derived ENS in vitro system is the most comprehensive one to date and furthers the potential for the use of this approach in the field while also providing useful guidelines. However, there are a few issues that need to be addressed, the main one regarding the assumptions adopted in the presented model. In the proposed models the authors assume that a) the same proliferation rate applies to glia and progenitors (line 613) and b) neurons die (lines 619-620) and they are the only cells in the model that do so. It has been previously shown that mature glia proliferate at a markedly reduced rate in comparison to ENS progenitors, so this parameter needs to be corrected. Currently, the assumption used is likely to result in lineage bias towards neurons as in the model glia will proliferate more than expected after differentiating. Furthermore, neurons are assumed to be quiescent and undergo cell death at a constant rate. Evidence supporting this assumption need to be provided here or the model should be adjusted accordingly (i.e. neuronal death being removed). Following the suggested adjustments, outcomes from the models should be compared anew with the existing literature from animal-based studies.

© 2026. Published by The Company of Biologists under the terms of the Creative Commons Attribution License (https://creativecommons.org/licenses/by/4.0/).

Additionally, please see some further comments below:

* Notch signalling inhibition section (starting line 233)/Fig.4: Consider clarifying further when referring to cell numbers vs cell proportions. Furthermore, in addition to discussing the increase in proportions of ENS cell types, please specify the cell types that decreased in proportion following Notch inhibition (e.g. mesenchymal-like cells?). Please present the proportional changes when ENS-only cells are considered/used as a denominator (i.e. excluding mesenchymal-like cells).
* Fig.2C: Keep the colours consistent between the individual channel panels and the merged channels panels
* Line 195/Fig.2D: The image presented does not clearly show neuronal cell bodies in the TUJ1 staining to support the statement that 'these glial cells were interspersed among TUJ1+ neurons'. Please replace with a more representative image if possible.
* Line 255/Fig.4: Comment on SOX10+ cell numbers at D22, as with the other cell types
* Fig.4A/B: Please clarify the 'relative to hPSCs' data/calculations in the figure legend and the methods section (i.e. was undifferentiated hPSCs cultured in parallel and processed simultaneously with the ENS cultures or is this referring to hPSC data collected prior to the experiments etc)
* Line 286/Fig.5D: The models do not appear to be significantly different. Consider rephrasing 'Despite this' to 'In agreement with this'.
* Fig.5F: consider including the differentiation rate gradient for all 4 models.
* Line 325: add the differentiation rater at D22 for the controls.
* Consider commenting on the impact of Notch inhibition in the generation of the different types of enteric neurons (including NOS+ neurons).

Reviewer 3

In this manuscript, the authors investigated the role of Notch signaling during enteric nervous system differentiation using a human pluripotent stem cell-based model. They showed that inhibition of Notch signaling using DAPT promoted both neuronal and glial differentiation while impaired migration. They also developed a mathematic model which suggests a lineage branching model with an early neuronal bias and a later glial bias. However, this part is mainly speculative. I have a few comments to help strengthen this manuscript.
1. The whole study relies on DAPT-mediated Notch inhibition. In Figure 3, HES1 is not affect by DAPT, raising questions about whether current DAPT treatment fully inhibits Notch signaling. The authors should test different doses of DAPT, and validate the key findings using other Notch signaling inhibitors, to demonstrate that the observed effects re not DAPT specific.
2. Figure 3 is interesting that continuous treatment of DAPT from day 9 to day 50 produces temporally dynamic and distinct effects on different Notch components. To further strength the mechanistic insights of this manuscript, the authors should perform Notch inhibition at different time windows, such as early (D13-22) vs late (D22-50). This could help determine whether Notch signaling has time dependent effect on ENS differentiation, as suggested by Figure 3. In addition, these experiments would also provide a stronger test of the proposed lineage branching model.
3. The mathematical modeling part is weak and mainly speculative. The predicted braching model is not supported by the experiments. The authors should provide direct experimental evidence that their hPSC-derived ENS model recapitulates this branching process and clarify how Notch inhibition alters lineage bias.
4. The scratch assay is interesting but require more extensive characterization. A scratch assay does not distinguish reduced migration from altered adhesion, proliferation, etc. Live cell imaging and cell tracking should be performed to directly examine the migration.
5. This study only used one hiPSC line. The authors should repeat key findings using at least one hESC line and another hiPSC line.

First revision

Author response to reviewers' comments

To the Editor

© 2026. Published by The Company of Biologists under the terms of the Creative Commons Attribution License (https://creativecommons.org/licenses/by/4.0/).

We are pleased to resubmit our manuscript entitled “Notch signalling governs human enteric nervous system progenitor dynamics” (MS ID#: dev.205775). We are grateful to the reviewers for the thoughtful and detailed evaluation of our manuscript. The revised version includes new data and text changes (highlighted in yellow), which address the issues raised.
Below is our point-to-point response to the suggestions/comments of the reviewers (in italics):

Reviewer 1

1. The authors themselves describe the culture conditions (that spheres are plating into) as "neurogenic conditions". How does this impact the conclusions drawn about both the differentiation trajectories in normal conditions, and the impact of Notch signalling? Can Notch inhibition drive neuronal and glial differentiation of vNCC/ENCC in the absence of neurogenic conditions (GDNF, AA)?

We thank the reviewer for raising this important point. Notch signalling inhibition has been shown to promote differentiation of neural progenitor entities in various distinct human pluripotent stem cell (hPSC) differentiation protocols (Borghese et al., 2010, Maury et al., 2015, Elkabetz et al., 2008). However, the dependence of Notch signalling on extrinsic cues appears to be context-dependent. hPSC-derived spinal motor neuron progenitors are capable of differentiating toward post mitotic motor neurons under the influence of the Notch inhibitor DAPT in the absence of retinoic acid and SHH agonists (Maury et al., 2015), while in hPSC-derived neural stem cells maintained in FGF2/EGF, Notch inhibition alone is insufficient to overcome the anti-differentiation effects of these growth factors(Borghese et al., 2010). In the context of enteric nervous system (ENS) development, there is evidence of functional crosstalk between GDNF/RET and Notch signalling. Aganglionosis-causing mutations in *RET*, which is activated by GDNF, result in dysregulation of Notch signalling components (Vincent et al., 2023, Windster et al., 2025), suggesting that these pathways interact during ENS development. Interestingly, GDNF has been reported to attenuate Notch signalling following transplantation of hPSC-derived neural progenitors in a spinal cord injury model (Khazaei et al., 2020) pointing to a conservation of this interaction across different systems.

Although it would be highly informative to determine whether Notch inhibition alone can drive neuronal and glial differentiation in the absence of GDNF and ascorbic acid, this question is difficult to address in our cultures. Day 9 spheres comprise a heterogeneous population containing vagal neural crest-derived ENS progenitors together with co-emerging non-ENS cell types, including mesenchymal cells (see main text and response below). These neighbouring cell populations are likely to contribute to Notch activation through juxtacrine signalling, making it difficult to isolate the effects of extrinsic GDNF signalling from endogenous cell-cell interactions. Addressing this question would therefore require purification of specific cellular subpopulations (e.g. by FACS) followed by optimisation of differentiation under defined culture conditions (+/-GDNF; +/-AA), which is beyond the scope of the present study. We agree, however, that dissecting the interplay between Notch signalling and extrinsic neurogenic cues represents an important avenue for future investigation. To acknowledge this limitation, we have added a statement to the Discussion (lines 472-476 in the new manuscript version) clarifying that our conclusions regarding the role of Notch signalling are restricted to the neurogenic differentiation conditions used in this study.

2. In Figure 3B, significant upregulation of DLL1/3/4 in the Notch inhibition conditions makes sense. But can the authors explain the significant upregulation of Notch 2/Jag 2 after DAPT treatment (at later timepoints)?

The simultaneous upregulation of *NOTCH2* and *JAG2* transcript levels at day 38 of differentiation is likely to reflect a combination of compensatory transcriptional feedback and developmental stage-specific remodelling of Notch receptor-ligand interactions as ENS cultures mature. Following sustained DAPT-mediated inhibition of Notch signalling, as evidenced by the persistent downregulation of the canonical target *HES5*, changes in the expression repertoire of Notch receptors and ligands may represent an adaptive response to altered pathway activity and evolving cell-cell communication within the differentiating cultures. In this context, the early induction of Delta-like ligands and the later emergence of *NOTCH2*/*JAG2* expression may signify the transition from a progenitor-enriched state towards a population containing maturing enteric neurons, glia and other non-ENS cell types with distinct Notch signalling requirements. Consistent with this interpretation, compensatory upregulation of Notch pathway components following pharmacological inhibition of Notch signalling has been reported in various different systems (e.g. (Reynolds et al., 2022, Campbell et al., 2016, Tsao et al., 2008).

© 2026. Published by The Company of Biologists under the terms of the Creative Commons Attribution License (https://creativecommons.org/licenses/by/4.0/).

3. Line 235-237: I think "...throughout the course of differentiation" slightly misrepresents the data. It suggests all transcripts upregulated across all (or most) time points, however most genes only significantly upregulated at 1-2 time points. Maybe rephrase (eg: "....at different time points throughout the course of differentiation".
We agree with the reviewer's suggestion and we have amended the text accordingly (line 244 in new manuscript version).

4. The scratch assay data is compelling. But I find it a little difficult to separate a direct effect on migration from the accelerated differentiation associated with DAPT treatment. Could this reduced wound closure be purely a symptom of less progenitors and more differentiation, rather than actual migration? Have you looked at expression of key migratory genes following DAPT treatment?
We have now performed additional analysis of live imaging data from the scratch assay, quantifying single-cell migration dynamics (new Figures 6G, S7). This revealed that DAPT-treated ENS cultures are marked by reduced migration velocity and, most prominently, defective migration directionality compared with DMSO-treated controls. These findings indicate that the delayed wound closure observed following Notch inhibition is not solely a consequence of changes in cellular composition or accelerated differentiation but is associated with an intrinsic impairment in the migratory behaviour of ENS cells. We have incorporated these new data into the revised manuscript and have clarified this interpretation in the Results and Discussion sections (Figures 6G, S7C/lines 418-424 and 489-502 in the new manuscript version). While we have not performed a comprehensive analysis of migration-associated gene expression, in our view, this single-cell tracking analysis provides a more direct functional assessment of migratory behaviour linked to the phenotype measured by the scratch assay.

5. One of the main conclusions (in the abstract) is that inhibition of Notch "...accelerates differentiation coincident with a shift toward increased gliogenesis". I don't see strong evidence from the biology of a shift toward glial fate. The modelling may suggest it, but I think if that's the only evidence then the language needs to be softened or the sentence clarified.
We agree, and our revised modelling (see response to Reviewer 2) now supports the reviewer's reading of the biology. Under the corrected model-in which the unidentified $SOX10^{-}/S100B^{-}/HuC/D^{-}$ population is treated as co-emerging rather than progenitor-derived, and glia no longer share the progenitor proliferation rate-we no longer find a shift toward a gliogenic bias. The inferred neuronal-versus-glial bias is essentially unchanged between control and DAPT (neuronal share ~ 0.74 in both), while the overall differentiation rate roughly doubles: Notch inhibition accelerates the production of both neurons and glia rather than redirecting fate toward glia. We have accordingly revised the Abstract to read "accelerates differentiation, coincident with the premature production of both neurons and glia" with matching changes to the Introduction, Results and Discussion.

- Line 90: Already a number of references included, but would consider adding McCallum et al 2020 (PMID: 32851974) as this directly addresses the role of Notch signalling in enteric neuro-gliogenesis (in zebrafish).
We thank the reviewer for this suggestion and have now included the McCallum et al. (2020) study in the revised manuscript (line 94 in new manuscript version).

- Figure 4: Please label the nearest neighbour quantification (bar graph) Figure 4J, for consistency.
We have now updated the labelling of the nearest-neighbour quantification bar graph in Figure 4J to ensure consistency with the other figure panels.

Reviewer 2
in the proposed models the authors assume that a) the same proliferation rate applies to glia and progenitors (line 613) and b) neurons die (lines 619-620) and they are the only cells in the model that do so. It has been previously shown that mature glia proliferate at a markedly reduced rate in comparison to ENS progenitors, so this parameter needs to be corrected. Currently, the assumption used is likely to result in lineage bias towards neurons as in the model glia will proliferate more than expected after differentiating. Furthermore, neurons are assumed to be quiescent and undergo cell death at a constant rate. Evidence supporting this assumption need to be provided here or the model should be adjusted accordingly (i.e. neuronal death being

© 2026. Published by The Company of Biologists under the terms of the Creative Commons Attribution License (https://creativecommons.org/licenses/by/4.0/).

removed). Following the suggested adjustments, outcomes from the models should be compared anew with the existing literature from animal-based studies.

We are grateful to the reviewer for these constructive comments on the model's assumptions, which prompted a revision of our modelling framework. We summarise the main changes below; the corresponding edits to the Methods, Results and new Figures 5, S4-6 are shown in the revised manuscript.

(1) Proliferation and death of glia and neurons. We agree that assuming a shared proliferation rate for progenitors and glia was not justified and, as the reviewer notes, would bias the inferred lineage balance towards neurons. In the revised model glia do not appreciably self-renew: mature enteric glia proliferate at a very low rate relative to progenitors (Laddach et al., 2023, Joseph et al., 2011), and this rate is not identifiable from our two time points, so we fix glial self-renewal to zero rather than sharing the progenitor rate. We have likewise removed the neuronal death term: enteric neurons are largely post-mitotic once formed (Morarach et al., 2021) and we have no evidence for substantial neuronal apoptosis over this window, so neurons are now treated as quiescent, neither dividing nor dying. This also fixes the model's rate gauge, as all rates are now expressed relative to the neuron compartment. Neurons and glia therefore neither proliferate nor die in the revised model.

(2) Identity of the unidentified population. While reviewing the model's assumptions, we determined that treating the $SOX10^{-}/S100B^{-}/HuC/D^{-}$ unidentified cells as a differentiation product of ENS progenitors was not well supported, since ENS progenitors are generally held to give rise only to neurons and glia. We have therefore reframed this population as a co-emerging population, most plausibly mesenchymal-like cells derived from day 6 vagal NC and/or cranial/cardiac NC contaminants (Bixby et al., 2002, Ling and Sauka-Spengler, 2019, Sabahi-Kaviani et al., 2026), that is present in the cultures but is not derived from the ENS progenitors, and that evolves with its own net rate. In the revised model progenitors give rise only to neurons and glia, with a single bias ρ setting the neuronal share of differentiation and $1 - \rho$ the glial share.

(3) Statistical inference. To make fuller use of the empirical data, we have replaced the summary-statistic Approximate Bayesian Computation scheme with a compositional likelihood evaluated directly on the field-level cell counts: a beta-binomial distribution for the HuC/D^{+} neuron field and a Dirichlet-multinomial distribution for the SOX10/S100B field, each conditioned on the observed field totals. This accounts explicitly for the substantial variability between biological replicates and does not depend on the unknown initial cell number. Posterior distributions are sampled by the No-U-Turn sampler (Hoffman and Gelman, 2014), with convergence assessed by the rank-normalised $\hat{R}$ statistic and the effective sample size (Vehtari, 2021).

(4) Comparison of posterior parameter estimates. Finally, we have replaced the Jensen-Shannon-divergence permutation test with a comparison based on posterior medians and credible intervals (Supplemental Table S1), since a permutation test applied to posterior samples primarily reflects the precision with which each posterior is estimated rather than the magnitude of any biological difference.

Following these adjustments, and as the reviewer requested, we have re-examined our conclusions against the animal literature. The revised analysis continues to indicate that Notch inhibition accelerates the overall rate of ENS progenitor differentiation, consistent with the premature neuronal differentiation and progenitor depletion reported in mouse (Okamura and Saga, 2008), while indicating that the neuronal-versus-glial bias is largely stable rather than switching towards a gliogenic fate. We have revised the Abstract, Results and Discussion accordingly.

Notch signalling inhibition section (starting line 233)/Fig.4: Consider clarifying further when referring to cell numbers vs cell proportions.

We have revised the text throughout the manuscript to clarify when we refer to changes in cell numbers versus changes in relative proportions.

Furthermore, in addition to discussing the increase in proportions of ENS cell types, please specify the cell types that decreased in proportion following Notch inhibition (e.g. mesenchymal-like cells?).

© 2026. Published by The Company of Biologists under the terms of the Creative Commons Attribution License (https://creativecommons.org/licenses/by/4.0/).

The SOX10⁻/S100B⁻/HuC/D⁻ unidentified population was indeed the only population whose proportion decreased following Notch inhibition, and our model quantifies this directly through the inferred net per-capita rate α_U, which is indistinguishable from zero in control but clearly negative under Notch inhibition. We have added text to the Results reporting these values alongside the corresponding changes in the other populations (lines 278-398 in new manuscript version).

Please present the proportional changes when ENS-only cells are considered/used as a denominator (i.e. excluding mesenchymal-like cells).
As requested, we now present proportions normalised to the ENS compartment (P+N+G) in the new Figure S6. We note that in the revised model the co-emerging population U is dynamically uncoupled from the ENS lineages, and the ENS-normalised proportions are independent of both α_U and P_0; the parameter estimates and model comparison are therefore unchanged, and the normalised presentation is obtained directly from the fitted trajectories. This presentation also removes the effect of the declining mesenchymal fraction, which differs between conditions (median U/T falls from 0.85 to 0.65 in control and from 0.84 to 0.27 under Notch inhibition), and makes clear that the change within the ENS compartment over D15-D22 is predominantly a shift from progenitors to glia rather than a change in neuronal share.

Fig.2C: Keep the colours consistent between the individual channel panels and the merged channels panels
We have updated Figure 2C to ensure colour consistency between panels.

Line 195/Fig.2D: The image presented does not clearly show neuronal cell bodies in the TUJ1 staining to support the statement that 'these glial cells were interspersed among TUJ1+ neurons'. Please replace with a more representative image if possible.
We have included higher magnification/additional new images that more clearly demonstrate the localisation of $SOX10^+$ cells among $TUJ1^+$ neurons/neuronal cell bodies (new Figures 2D and S2C). These changes better support the description in the text.

Line 255/Fig.4: Comment on SOX10+ cell numbers at D22, as with the other cell types
We have updated the text in the Results section to include a description of the $SOX10^+$ cell numbers at day 22 (line 262 in new manuscript version).

Fig.4A/B: Please clarify the 'relative to hPSCs' data/calculations in the figure legend and the methods section (i.e. was undifferentiated hPSCs cultured in parallel and processed simultaneously with the ENS cultures or is this referring to hPSC data collected prior to the experiments etc)
The undifferentiated hPSC samples used for the “relative to hPSCs” calculations were collected as cell pellets immediately prior to initiating differentiation, representing the starting population used to generate the ENS cultures. RNA was extracted from these samples and analysed alongside the differentiated samples within the same RT-qPCR experiment. We have updated the text in the Methods section to clarify this experimental design (lines 572-576 in new manuscript version).

Line 286/Fig.5D: The models do not appear to be significantly different. Consider rephrasing 'Despite this' to 'In agreement with this'.
We have amended the text to remove “Despite this”.

Fig.5F: consider including the differentiation rate gradient for all 4 models.
To clarify, a differentiation rate gradient is only defined as a fitted parameter for two of the four models. All four models contain the baseline differentiation rate parameter (k0) (see Eqs. 5 and 7), but the two constant-rate models do not contain the linear time-dependent term (k1). The same distinction applies to the corresponding bias terms (Eqs. 6 and 8).

Line 325: add the differentiation rater at D22 for the controls.
We have added the inferred differentiation rates at D22 for the DMSO condition as requested.

Consider commenting on the impact of Notch inhibition in the generation of the different types of enteric neurons (including NOS+ neurons).
The reviewer raises an interesting point. In the current study, we focused on the role of Notch signalling in regulating ENS progenitor dynamics/cell fate decisions consistent with previous studies

© 2026. Published by The Company of Biologists under the terms of the Creative Commons Attribution License (https://creativecommons.org/licenses/by/4.0/).

in animal models (Okamura and Saga, 2008, Mead and Yutzey, 2012, Kuil et al., 2023, Taylor et al., 2007). The potential role of Notch signalling in specifying individual enteric neuronal subtypes remains comparatively unexplored. To our knowledge, only one study has touched on this question, reporting an increase in nitric oxide synthase (NOS)-expressing neurons following DAPT treatment of mouse ENS neurospheres (Theocharatos et al., 2013) Further investigation of this aspect of Notch function represents an important future direction, particularly given the reported differential expression of Notch pathway components among enteric neuronal populations(Morarach et al., 2021, Sander et al., 2003). This suggests that distinct Notch receptor-ligand interactions may contribute to enteric neuronal subtype diversification. We have added a comment on this in the Discussion section (lines 455-460 in the new manuscript version).

Reviewer 3

1. The whole study relies on DAPT-mediated Notch inhibition. In Figure 3, HES1 is not affect by DAPT, raising questions about whether current DAPT treatment fully inhibits Notch signaling. The authors should test different doses of DAPT, and validate the key findings using other Notch signaling inhibitors, to demonstrate that the observed effects re not DAPT specific.

The selective dramatic reduction of *HES5* but not *HES1* transcripts following DAPT treatment is consistent with previous studies demonstrating differential sensitivity of Notch target genes to pathway inhibition. In various developmental contexts, including hPSC-derived neural stem cells and lung progenitor systems, *HES5* has been shown to be strongly reduced following pharmacological Notch inhibition, whereas *HES1* expression is less affected (Borghese et al., 2010, Tsao et al., 2008). This likely reflects that the fact that, although both genes can be regulated by Notch signalling, *HES1* expression is also controlled by Notch-independent pathways (Katoh and Katoh, 2007, Wall et al., 2009).

To further address this point, we have now included additional data examining the effect of increasing DAPT concentrations (10–50 µM) on *HES1*/*HES5* expression (new Figure S3A, B; lines 214-216 in new manuscript version). These experiments demonstrated a comparable effect of DAPT treatment across the tested concentrations, with no additional reduction at higher doses, supporting the use of 10 µM as an effective concentration for our experiments while minimising potential off-target effects associated with higher doses. Although we did not repeat the experiments using alternative Notch inhibitors in the present study, we have previously shown that pharmacological inhibition of Notch signalling using DAPT produces comparable effects to the γ-secretase inhibitor LY411575 (Cooper et al., 2024). Together with the dose-response analysis presented here and the consistent suppression of the canonical Notch target *HES5*, these findings support that the phenotypes observed are due to inhibition of Notch pathway activity rather than a DAPT-specific effect.

2. Figure 3 is interesting that continuous treatment of DAPT from day 9 to day 50 produces temporally dynamic and distinct effects on different Notch components. To further strength the mechanistic insights of this manuscript, the authors should perform Notch inhibition at different time windows, such as early (D13-22) vs late (D22-50). This could help determine whether Notch signaling has time dependent effect on ENS differentiation, as suggested by Figure 3. In addition, these experiments would also provide a stronger test of the proposed lineage branching model.

We thank the reviewer for this thoughtful suggestion about the temporal impact of Notch inhibition. However, addressing this issue would require the establishment and optimisation of a substantially expanded experimental framework. In particular, initiating DAPT treatment at later stages of differentiation would necessitate re-optimisation of our phenotypic analyses, as the cultures become increasingly dense and structurally complex over time, making reliable single-cell quantification of neuronal and glial populations by immunofluorescence considerably more challenging. Moreover, the resulting datasets would require independent modelling to distinguish the effects of transient versus continuous Notch inhibition on differentiation kinetics and lineage allocation.

The primary objective of the present study was to define the role of sustained Notch inhibition during the early stages of human ENS progenitor differentiation, when cell fate decisions are first established. While our data reveal temporally dynamic changes in the expression of Notch pathway components during in the presence/absence of continuous DAPT treatment, we agree that determining whether discrete developmental windows exhibit differential sensitivity to Notch inhibition is an important question that extends beyond the scope of the current study. We have

© 2026. Published by The Company of Biologists under the terms of the Creative Commons Attribution License (https://creativecommons.org/licenses/by/4.0/).

therefore added a statement to the Discussion highlighting this as an important direction for future work (lines 484-487 in new manuscript version).

3. The mathematical modeling part is weak and mainly speculative. The predicted braching model is not supported by the experiments. The authors should provide direct experimental evidence that their hPSC-derived ENS model recapitulates this branching process and clarify how Notch inhibition alters lineage bias.
We firmly disagree that the modelling is "weak and mainly speculative." It is not a speculative construct set alongside the experiments but an inference framework fitted directly to them (see also response to Reviewers 1 and 2): we infer differentiation rates and lineage biases from the measured SOX10/S100B and HuC/D proportions (Figure 5E, G) using a compositional likelihood on the raw counts, sampled by Hamiltonian Monte Carlo with full convergence diagnostics and model comparison by cross-validation. The parameters are constrained by the data, not assumed, and the posterior predictive checks (Figures 5D, 5J, S4, S5) confirm the models reproduce the observed proportions. The analysis is also explicit about its limits - we state that the candidate scenarios cannot be distinguished at current resolution, rather than asserting a single "best" model.

On the "branching" concern: we do not claim the modelling independently establishes a branching hierarchy; that framework is from prior mouse single-cell work (Laddach et al., 2023) and we use it only to frame how rate and bias evolve. Our revised analysis does not support a strong gliogenic switch - Notch inhibition roughly doubles the overall differentiation rate while leaving the neuronal vs glial bias essentially unchanged - and we have tempered the branching and gliogenic language accordingly. This answers the reviewer's question directly: within this window Notch inhibition alters the pace of differentiation, not the fate split.

Importantly, this key conclusion is model-free: the ratio of committed cells to progenitors, (N+G)/P - independent of absolute scale and of the unidentified population - approximately doubles under Notch inhibition, giving direct experimental evidence of accelerated commitment. The experimental evidence the reviewer requests is thus the very data the model is fitted to: the qPCR and immunofluorescence time courses showing neurons and glia emerging from SOX10$^+$ progenitors (Figures 1, 2, 4). The modelling is a quantitative synthesis of these experiments, not a claim made in their absence.

4. The scratch assay is interesting but require more extensive characterization. A scratch assay does not distinguish reduced migration from altered adhesion, proliferation, etc. Live cell imaging and cell tracking should be performed to directly examine the migration.
We have included additional data from analysis of live imaging/scratch assay experiments showing that DAPT-treated ENS cultures exhibit reduced migration velocity and impaired migration directionality compared with DMSO-treated controls (Figures 6G, S7C in the new manuscript version). This indicates that the delayed wound closure observed following Notch inhibition is a consequence of an intrinsic defect in migratory behaviour rather than changes in cell adhesion, proliferation or differentiation state. Please see our response to Reviewer 1 (Comment #4) for further details.

5. This study only used one hiPSC line. The authors should repeat key findings using at least one hESC line and another hiPSC line.
The ability of DAPT treatment to promote ENS differentiation has previously been demonstrated using multiple independent human pluripotent stem cell (hPSC) lines, including both human embryonic stem cell (hESC) and induced pluripotent stem cell (hiPSC) lines (Cooper et al., 2024, Frith et al., 2020, Gogolou et al., 2021), supporting the reproducibility of this effect. To further strengthen the present study, we have now included additional qPCR and immunofluorescence data showing the efficient differentiation of a second hPSC line, the WA09-derived SOX10-GFP reporter hESC line (Chambers et al., 2012), using our DAPT-based differentiation protocol (new Figure S2, lines 198-200 in new manuscript version).

References
BIXBY, S., KRUGER, G. M., MOSHER, J. T., JOSEPH, N. M. & MORRISON, S. J. 2002. Cell-intrinsic differences between stem cells from different regions of the peripheral nervous system regulate the generation of neural diversity. *Neuron,* 35, 643-56.

© 2026. Published by The Company of Biologists under the terms of the Creative Commons Attribution License (https://creativecommons.org/licenses/by/4.0/).

BORGHESE, L., DOLEZALOVA, D., OPITZ, T., HAUPT, S., LEINHAAS, A., STEINFARZ, B., KOCH, P., EDENHOFER, F., HAMPL, A. & BRUSTLE, O. 2010. Inhibition of notch signaling in human embryonic stem cell-derived neural stem cells delays G1/S phase transition and accelerates neuronal differentiation in vitro and in vivo. *Stem Cells,* 28, 955-64.
CAMPBELL, D. P., CHRYSOSTOMOU, E. & DOETZLHOFER, A. 2016. Canonical Notch signaling plays an instructive role in auditory supporting cell development. *Sci Rep,* 6, 19484.
CHAMBERS, S. M., QI, Y., MICA, Y., LEE, G., ZHANG, X. J., NIU, L., BILSLAND, J., CAO, L., STEVENS, E., WHITING, P., SHI, S. H. & STUDER, L. 2012. Combined small-molecule inhibition accelerates developmental timing and converts human pluripotent stem cells into nociceptors. *Nat Biotechnol,* 30, 715-20.
COOPER, F., SOUILHOL, C., HASTON, S., GRAY, S., BOSWELL, K., GOGOLOU, A., FRITH, T. J. R., STAVISH, D., JAMES, B. M., BOSE, D., KIM DALE, J. & TSAKIRIDIS, A. 2024. Notch signalling influences cell fate decisions and HOX gene induction in axial progenitors. *Development,* 151.
ELKABETZ, Y., PANAGIOTAKOS, G., AL SHAMY, G., SOCCI, N. D., TABAR, V. & STUDER, L. 2008. Human ES cell-derived neural rosettes reveal a functionally distinct early neural stem cell stage. *Genes Dev,* 22, 152-65.
FRITH, T. J. R., GOGOLOU, A., HACKLAND, J. O. S., HEWITT, Z. A., MOORE, H. D., BARBARIC, I., THAPAR, N., BURNS, A. J., ANDREWS, P. W., TSAKIRIDIS, A. & MCCANN, C. J. 2020. Retinoic Acid Accelerates the Specification of Enteric Neural Progenitors from In-Vitro-Derived Neural Crest. *Stem Cell Reports,* 15, 557-565.
GOGOLOU, A., FRITH, T. J. R. & TSAKIRIDIS, A. 2021. Generating Enteric Nervous System Progenitors from Human Pluripotent Stem Cells. *Curr Protoc,* 1, e137.
HOFFMAN, M. D. & GELMAN, A. 2014. The No-U-Turn Sampler: Adaptively Setting Path Lengths in Hamiltonian Monte Carlo. *Journal of Machine Learning Research,* 15, 1593-1623.
JOSEPH, N. M., HE, S., QUINTANA, E., KIM, Y. G., NUNEZ, G. & MORRISON, S. J. 2011. Enteric glia are multipotent in culture but primarily form glia in the adult rodent gut. *J Clin Invest,* 121, 3398-411.
KATOH, M. & KATOH, M. 2007. Integrative genomic analyses on HES/HEY family: Notch-independent HES1, HES3 transcription in undifferentiated ES cells, and Notch-dependent HES1, HES5, HEY1, HEY2, HEYL transcription in fetal tissues, adult tissues, or cancer. *Int J Oncol,* 31, 461-6.
KHAZAEI, M., AHUJA, C. S., NAKASHIMA, H., NAGOSHI, N., LI, L., WANG, J., CHIO, J., BADNER, A., SELIGMAN, D., ICHISE, A., SHIBATA, S. & FEHLINGS, M. G. 2020. GDNF rescues the fate of neural progenitor grafts by attenuating Notch signals in the injured spinal cord in rodents. *Sci Transl Med,* 12.
KUIL, L. E., KAKIAILATU, N. J. M., WINDSTER, J. D., BINDELS, E., ZINK, J. T. M., VAN DER ZEE, G., HOFSTRA, R. M. W., SHEPHERD, I. T., MELOTTE, V. & ALVES, M. M. 2023. Unbiased characterization of the larval zebrafish enteric nervous system at a single cell transcriptomic level. *iScience,* 26, 107070.
LADDACH, A., CHNG, S. H., LASRADO, R., PROGATZKY, F., SHAPIRO, M., ERICKSON, A., SAMPEDRO CASTANEDA, M., ARTEMOV, A. V., BON-FRAUCHES, A. C., AMANITI, E. M., KLEINJUNG, J., BOEING, S., ULTANIR, S., ADAMEYKO, I. & PACHNIS, V. 2023. A branching model of lineage differentiation underpinning the neurogenic potential of enteric glia. *Nat Commun,* 14, 5904.
LING, I. T. C. & SAUKA-SPENGLER, T. 2019. Early chromatin shaping predetermines multipotent vagal neural crest into neural, neuronal and mesenchymal lineages. *Nat Cell Biol,* 21, 1504-1517.
MAURY, Y., COME, J., PISKOROWSKI, R. A., SALAH-MOHELLIBI, N., CHEVALEYRE, V., PESCHANSKI, M., MARTINAT, C. & NEDELEC, S. 2015. Combinatorial analysis of developmental cues efficiently converts human pluripotent stem cells into multiple neuronal subtypes. *Nat Biotechnol,* 33, 89-96.
MEAD, T. J. & YUTZEY, K. E. 2012. Notch pathway regulation of neural crest cell development in vivo. *Dev Dyn,* 241, 376-89.
MORARACH, K., MIKHAILOVA, A., KNOFLACH, V., MEMIC, F., KUMAR, R., LI, W., ERNFORS, P. & MARKLUND, U. 2021. Diversification of molecularly defined myenteric neuron classes revealed by single-cell RNA sequencing. *Nat Neurosci,* 24, 34-46.
OKAMURA, Y. & SAGA, Y. 2008. Notch signaling is required for the maintenance of enteric neural crest progenitors. *Development,* 135, 3555-65.

© 2026. Published by The Company of Biologists under the terms of the Creative Commons Attribution License (https://creativecommons.org/licenses/by/4.0/).

REYNOLDS, S. D., HILL, C. L., ALSUDAYRI, A., LALLIER, S. W., WIJERATNE, S., TAN, Z. H., CHIANG, T. & CORMET-BOYAKA, E. 2022. Assemblies of JAG1 and JAG2 determine tracheobronchial cell fate in mucosecretory lung disease. *JCI Insight,* 7.
SABAHI-KAVIANI, R., GOGOLOU, A., SOUILHOL, C., VAN DER KROEG, M., KUSHNER, S. A., DE VRIJ, F. M. S., TSAKIRIDIS, A. & LUTTGE, R. 2026. Nervous System-on-Chip: Innovative Microfluidic Platform to Compartmentalize hiPSC-Derived Neural Networks. *Micromachines (Basel),* 17.
SANDER, G. R., BROOKES, S. J. & POWELL, B. C. 2003. Expression of Notch1 and Jagged2 in the enteric nervous system. *J Histochem Cytochem,* 51, 969-72.
TAYLOR, M. K., YEAGER, K. & MORRISON, S. J. 2007. Physiological Notch signaling promotes gliogenesis in the developing peripheral and central nervous systems. *Development,* 134, 2435-47.
THEOCHARATOS, S., WILKINSON, D. J., DARLING, S., WILM, B., KENNY, S. E. & EDGAR, D. 2013. Regulation of progenitor cell proliferation and neuronal differentiation in enteric nervous system neurospheres. *PLoS One,* 8, e54809.
TSAO, P. N., CHEN, F., IZVOLSKY, K. I., WALKER, J., KUKURUZINSKA, M. A., LU, J. & CARDOSO, W. V. 2008. Gamma-secretase activation of notch signaling regulates the balance of proximal and distal fates in progenitor cells of the developing lung. *J Biol Chem,* 283, 29532-44.
VEHTARI, A., GELMAN, A., SIMPSON, D., CARPENTER, B., BÜRKNER, P-C. 2021. Rank-Normalization, Folding, and Localization: An Improved R for Assessing Convergence of MCMC (with Discussion). *Bayesian Anal.* , 16, 667-718.
VINCENT, E., CHATTERJEE, S., CANNON, G. H., AUER, D., ROSS, H., CHAKRAVARTI, A. & GOFF, L. A. 2023. Ret deficiency decreases neural crest progenitor proliferation and restricts fate potential during enteric nervous system development. *Proc Natl Acad Sci U S A,* 120, e2211986120.
WALL, D. S., MEARS, A. J., MCNEILL, B., MAZEROLLE, C., THURIG, S., WANG, Y., KAGEYAMA, R. & WALLACE, V. A. 2009. Progenitor cell proliferation in the retina is dependent on Notch-independent Sonic hedgehog/Hes1 activity. *J Cell Biol,* 184, 101-12.
WINDSTER, J. D., KAKIAILATU, N. J. M., KUIL, L. E., ANTANAVICIUTE, A., SACCHETTI, A., MACKENZIE, K. C., PEULEN-ZINK, J., KAN, T. W., BINDELS, E., DE PATER, E., DOUKAS, M., VAN DEN BOSCH, T. P. P., YOUSEFI, S., BARAKAT, T. S., MEEUSSEN, C., SLOOTS, P., WIJNEN, R. M. H., PARIKH, K., BOESMANS, W., MELOTTE, V., HOFSTRA, R. M. W., SIMMONS, A. & ALVES, M. M. 2025. Human Enteric Glia Diversity in Health and Disease: New Avenues for the Treatment of Hirschsprung Disease. *Gastroenterology,* 168, 965-979 e12.

Second decision letter

MS ID#: dev.205775R1

MS TITLE: Notch signalling governs human enteric nervous system progenitor dynamics

AUTHORS: Anestis Tsakiridis; Antigoni Gogolou; Nikolas Stefanidis; Fay Cooper; Guillaume Blin; Stanley E. Strawbridge; Alexander G. Fletcher

Dear Dr Tsakiridis,

I am happy to tell you that your manuscript has been accepted for publication in Development, pending our standard publication integrity checks.

Reviewer 1

SUGGESTIONS TO AUTHORS

The authors have thoroughly addressed my concerns. The addition of live cell tracking for migratory dynamics is much welcomed!

Reviewer 3

The authors have fully addressed my comments, and I recommend acceptance.

© 2026. Published by The Company of Biologists under the terms of the Creative Commons Attribution License (https://creativecommons.org/licenses/by/4.0/).

© 2026. Published by The Company of Biologists under the terms of the Creative Commons Attribution License (https://creativecommons.org/licenses/by/4.0/).
